# Supplementary material for: The R.O.A.D. to clinical trial emulation
Source: Res Sq. 2025 Mar 19:rs.3.rs-5576146. Preprint. [Version 1] doi: 10.21203/rs.3.rs-5576146/v1 (PMC11957197; doi:10.21203/rs.3.rs-5576146/v1)
Supplement: Supplement 1 [file NIHPPrs5576146v1-supplement-1.pdf]

5 Supplementary Material

Figure S1 – Recurrence-free survival for patients defined by node 2 of the first OPT, in (A) the emulated cohort and (B) the RCT cohort

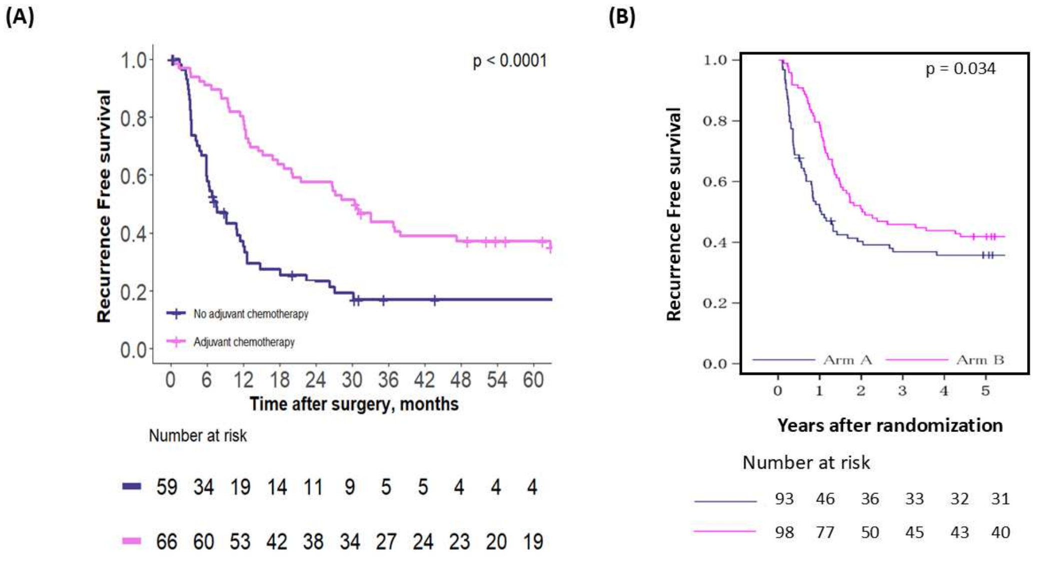

Figure S2 – Recurrence-free survival for patients defined by node 5 of the first OPT, in (A) the emulated cohort and (B) the RCT cohort

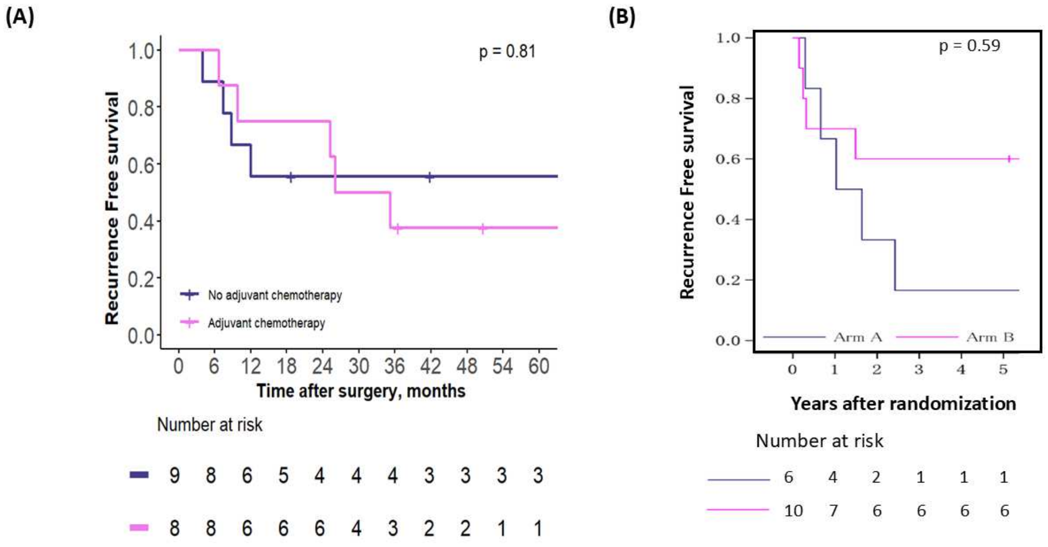

Figure S3 – Recurrence-free survival for patients defined by node 7 of the first OPT, in (A) the emulated cohort and (B) the RCT cohort

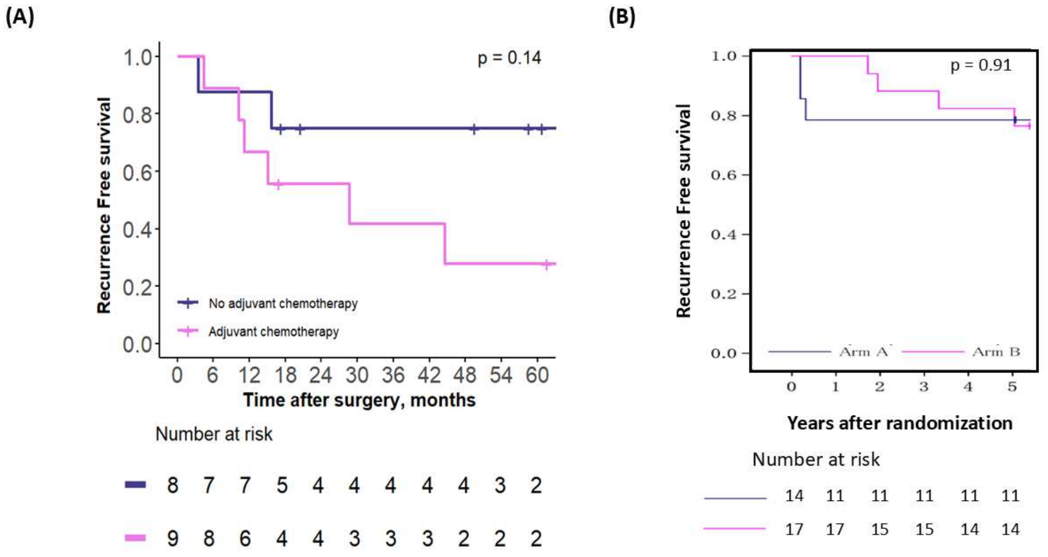

Figure S4 – Recurrence-free survival for patients defined by node 8 of the first OPT, in (A) the emulated cohort and (B) the RCT cohort

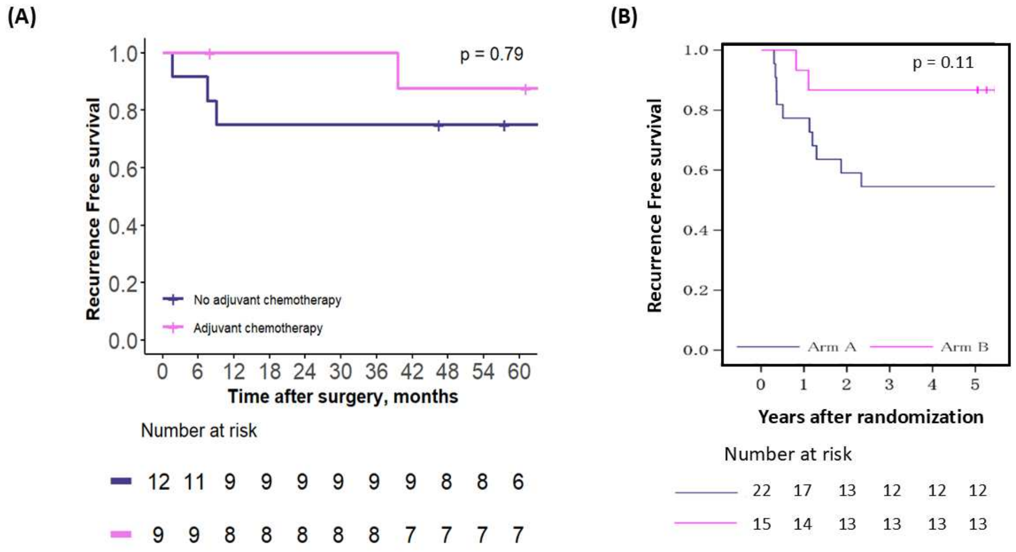

Figure S5 – Recurrence-free survival for patients defined by node 9 of the first OPT, in (A) the emulated cohort and (B) the RCT cohort

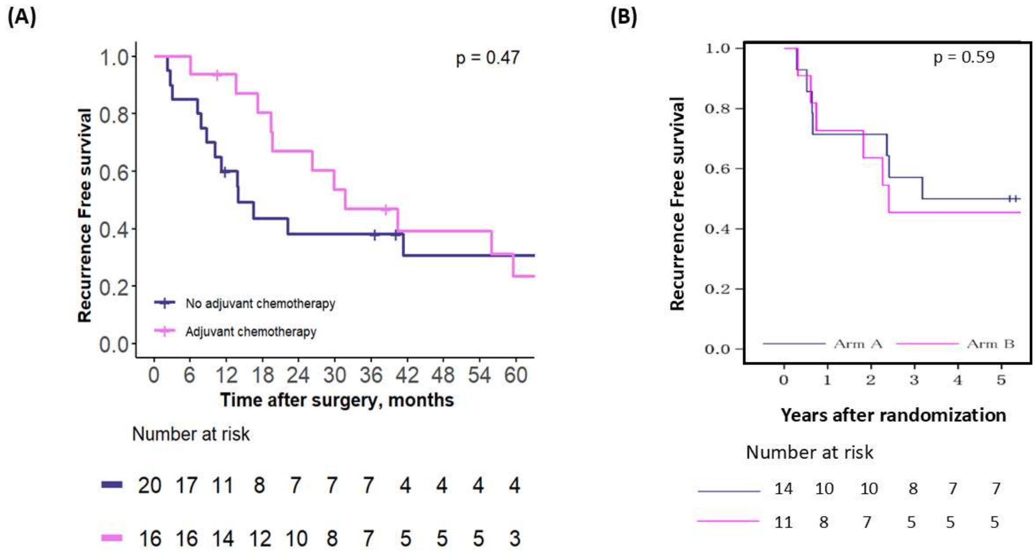

Figure S6 – Recurrence-free survival for patients defined by node 2 of the second OPT, in (A) the emulated cohort and (B) the RCT cohort

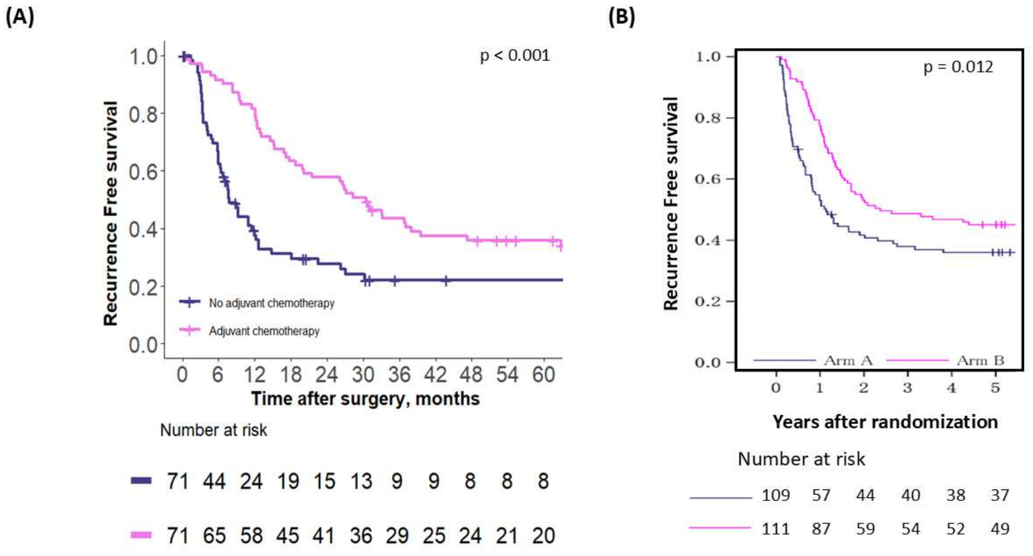

Figure S7 – Recurrence-free survival for patients defined by node 5 of the second OPT, in (A) the emulated cohort and (B) the RCT cohort

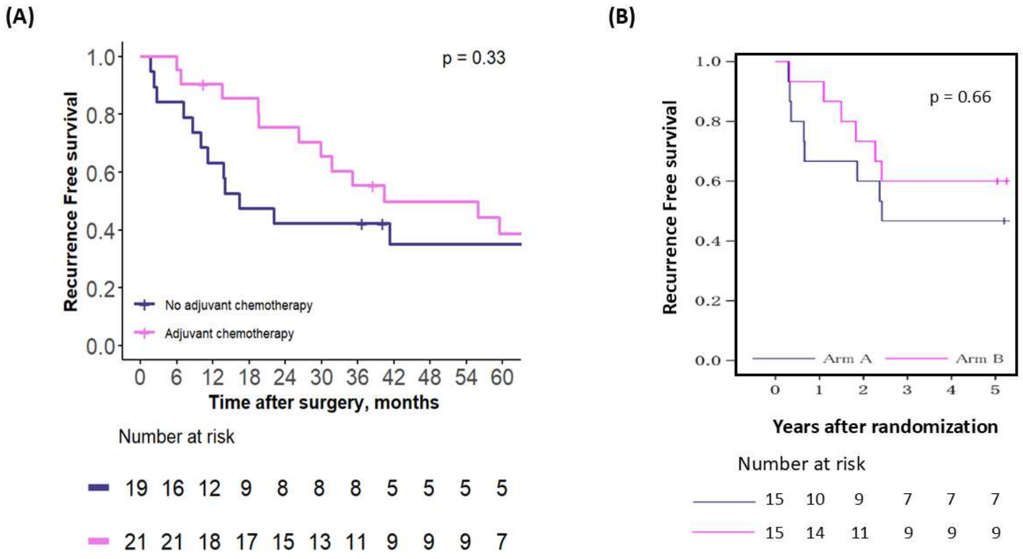

Table S1 – The median MSK CRS and JHH GAME scores for the nodes of the first OPT.

Node	Surgery alone	Adjuvant chemotherapy	P-value
**Node 2 (mean, SD)**			
MSK CRS	2.00[1.00, 3.00]	2.00[1.00, 3.00]	0.177
JHH GAME	1.00[1.00, 2.00]	2.00[1.00, 3.00]	0.051
**Node 5 (mean, SD)**			
MSK CRS	1.00[0.00, 1.00]	1.00[0.00, 1.00]	0.838
JHH GAME	1.00[1.00, 2.00]	1.00[1.00, 2.00]	0.755
**Node 7 (mean, SD)**			
MSK CRS	1.00[0.50, 1.00]	0.50[0.00, 1.00]	0.414
JHH GAME	1.00[0.00, 1.50]	1.00[0.75, 1.25]	0.624
**Node 8 (mean, SD)**			
MSK CRS	1.00[0.00, 1.00]	0.00[0.00, 1.00]	0.134
JHH GAME	2.00[1.00, 2.00]	2.00[1.00, 2.00]	0.613
**Node 9 (mean, SD)**			
MSK CRS	1.00[1.00, 1.00]	1.00[1.00, 1.00]	0.449
JHH GAME	2.00[1.50, 2.00]	2.00[1.00, 3.00]	0.512

The medians were compared using the Mann-Whitney U test; IQR: interquartile range
* The Memorial Sloan Kettering (MSK) Clinical Risk Score (CRS) for colorectal liver metastases (CRLM), and the Johns Hopkins Hospital (JHH) Genetic And Morphological Evaluation (GAME) score for CRLM.

Table S2 – The median MSK CRS and JHH GAME scores for the nodes of the second OPT.

Node	Surgery alone	Adjuvant chemotherapy	P-value
**Node 2 (median, [IQR])**			
MSK CRS	2.00[1.00, 3.00]	2.00[1.00, 3.00]	0.177
JHH GAME	1.00[1.00, 2.00]	2.00[1.00, 3.00]	0.051
**Node 4 (median, [IQR])**			
MSK CRS	1.00[0.25, 1.00]	0.00[0.00, 1.00]	0.061
JHH GAME	1.00[0.25, 2.00]	1.00[1.00, 2.00]	0.560
**Node 5 (median, [IQR])**			
MSK CRS	1.00[0.00, 1.00]	1.00[1.00, 1.00]	0.692
JHH GAME	2.00[1.00, 2.00]	2.00[1.00, 3.00]	0.661

The medians were compared using the Mann-Whitney U test; IQR: interquartile range
* The Memorial Sloan Kettering (MSK) Clinical Risk Score (CRS) for colorectal liver metastases (CRLM), and the Johns Hopkins Hospital (JHH) Genetic And Morphological Evaluation (GAME) score for CRLM.
